# A Canadian Dentist in Germany

**Published:** 1877-09

**Authors:** WM. R. Patton

**Affiliations:** Cologne, Germany.


### ARTICLE II.

### *A Canadian Dentist in Germany.*

BY WM. R. PATTON, D. D. S., COLOGNE, GERMANY.

Through some years experience of dentistry in a German centre, a few observations on the practice of our profession may not prove totally uninteresting to some of your readers —for among the very large majority of those with whom I came in contact during my last visit to the States and Canada, a decided lack of knowledge was shown relative to Continental practice and theory, combined with as decided a thirst for information. Our Continental colleagues do not stand so high in regard to operative work as the representatives of our specialty on the other side of the Atlantic, and this may be easily accounted for by the want of practical study in such institutions, devoted solely to that purpose, as the American colleges offer to the dental aspirant.

Until but lately, comparatively speaking, the German dentist has been struggling against the same disadvantages that the American suffered many years ago, viz: the acquiring of the knowledge they sought for, through the office of business of some practictioners in the capacity of drudge or assistant, a knowledge charily imparted through vague fears of later competition, being his only means of advancement.

Notwithstanding the foundation during the last few years of dental chairs in Berlin and Leipsig, etc., they are proved to be more theoretical than practical, and there is a general feeling, which is now expressing itself, from the leading class of the profession, for improvement.

General convocations of dentists, provincial and local, take place mostly yearly, in the American style, when dental subjects are discussed.

That a precedence is taken by American dentists on the continent, could be practically shown by naming the leading practitioners in most cities of large population: however as it is not my intention to give cause for rival comparison I will rigidly abstain from any thing verging on personality, merely observing in the interest of the profession—that dentistry on the Continent as represented by Americans, can claim the highest practical intelligence—also that there are native Continental practitioners whose writings as well as researches in the dental field have made their names known not only to Europe but America.

Refering to what I have remarked about the general lack of dental education on the Continent, I must in justice say that an annual increase occurs in the number of those who visit America from these shores in search of improvement, and who on their return seldom fail to meet with a just appreciation of their merits.

I am sorry to state however that the larger number remain but for one course, and having picked up somewhat at the practical manipulation, do not thoroughly perfect themselves, but return to compete on a firmer groundwork with the colleague who has not been capable of the same advantages.

American dentistry is represented as a body by the *American Dental Society of Europe*, the last meeting of which was held in Paris. Through absence, (being then in Canada) I cannot give you an account of that convention. The topics treated however, are of the same standard and general character of those discussed at similar assemblages in the United States; for example the list of essays for this summer embrace:

(1) Irregularities of the Teeth, cause and treatment.

(2) Cause of " " " "

(3) Is it desirable to separate Operative and Mechanical Dentistry?

(4) National types of Teeth.

(5) What materials are best for filling and preserving the teeth between the ages of twelve and fourteen ?

I hope to render you a good account of European opinion on these subjects.

As far as Germany is concerned the profession is represented by various societies.

Central Verein Deutscher Zahnarzte—

Gesellschaft Berliner Zahnarzte—

also societies in Frankfort a Main—Austria and Schleswig Holstein—these hold their meetings I believe annually. The American dentist excels in operating over his Continental colleague for the reason mentioned, but as mechanical workmen they can fully compete with their American bretheren. I have seen plate work of the highest standard, that had been in use for years, and showing skill equal to that of more modern date.

Still rubber has, as in the States, worked its evil influences upon the character of mechanical dentistry, and plate work (gold, etc.) by the many, may be considered a lost art—rubber, rubber, whole sets and partial, is the order of the day and at prices equal to the $8.00 sets of the celebrated American avenue men, and the reasons therefor are the same as those which cast a slur on the profession, even in the land of its fruition, the competition of merely mechanical men, who offer the public by every means in their power, swindling advertisements, etc., the benefits of cheap dentistry.

Fillings are made of Gold, Tin, Amalgam and the plastic preparations, the two latter being used to an extensive degree, and consequent detriment of many organs, capable of being well preserved through nobler manipulation.

Practical education is not alone necessary for the majority of German dentists, but also for the patients who are not universally elevated to the proper appreciation of artistic dentistry, who accustomed to submit to an operation which may necessarily occupy from one to three hours duration, and finally reward with a corresponding remuneration ; the only exceptions being the highest circle of society who really do take an interest in conservative dentistry. American dentistry has to struggle against the evil effect of cheap and consequently quick and imperfect work, the victimized becoming obstinate disbelievers in the worth of operative work, until the prejudice has been fairly eradicated by the lasting influences of some good conservative practitioner. Amalgam plays the greatest role amongst filling materials, and no wonder, for when the large majority are not educated to a proper remuneration the class of work must correspondingly balance the scale.

American dentists as educated operators receive the highest return for their labor, and are really appreciated when once their name and worth are known and spread through their abilities. That there are native professional men of the highest capacity, whose services are highly thought of there is no doubt, but they are in a minority to those, whose operations are not worth the small fees paid for them.

That the great question of dental education is causing thought in Europe as well as in America, the following article will prove to your readers—and probably will give a better idea of dentistry in Germany—than anything I could indite; for published in the German quarterly, edited by Dr. RobBaume, of Berlin—the organ of the Central German Society, it is a thoroughly German expression. I will translate it as literally as possible.

The Dental Tuition Question.—*By Dr. G. von Langsdorff.*—It is exhilarating to read, how on this and the other side of the ocean the question of elevating our dental standing, through bringing up qualified—that means theoretically practical educated Dentists, has become a subject for general earnest counsel.

For example, it is very easily said : *Only proper and able schools of culture meet our aim—or a gymnasial education as a ground work must be present ;—or Dental sur-*

*gery must be practised before all as a specialty ; or a dental candidate must be as fully capable of passing his medical and surgical examinations as his dental ;* and such like.

By reading and after thought over all there remain various propositions. I have formed by degrees the following code for dental education, which I now place before my readers.

I believe I can make my opinion clearest, when I undertake to throw light on the education question from *negative and positive* sides.

(1) The negative side—that is how Dentists should not be brought up, is now-a days undoubtedly the universal method.

We can presume that we have in Germany, at least a thousand practitioners, officiating as Dentists, that are carrying out the duties that can be claimed from a regular Dentist. Where have the majority of these thousand received their education ? Answer : Through having been one or more years with Dentists who for some few hundred shillings have taught them the most needful of that they themselves know.

Or, they are those who having worked here and there as Mechanics for Dentists, have gradually flattered themselves into the belief of their capability to practise self dependently the same business. Through power of the law which gives liberty of exercising any craft, (Gewerbe freiheit) they are allowed to make artificial sets; and as they stand under no control whatever so they extract and fill teeth and perform all other oral operations, as if they were *qualified Dentists* —with the only difference that they do not call themselves *Dentists*—but * *Tooth mechinists—Tooth practitioners—Tooth Artists—Technical dentist*, etc. This is then the class or "colleagues" through whom our calling is so much discredited : for these persons spoil and extract more teeth than they can ever be answerable for.

Or, there are Barber surgeons—Chemists—Apothecaries,

* **Tahntechniker—Tahnpractikant—Tahnkinstler—Techniker Tahnarz.**

—Watchmakers—Goldsmiths and other tradesmen who appropriate so far as they consider needful the technical work, * then practise every thing connected with the dental department. Solitary cases where persons have raised themselves through inate energy and assiduity must be regarded as exceptional.

Or again, there are medical students and even medical men who all at once get the idea into their heads to practise dentistry.—To make a rubber set of Teeth is soon learnt and that accomplished, they flatter themselves they are first class dentists. They are probably not in a position to faultlessly perform the simplest gold filling and yet they condescendingly look down on their colleagues, write themselves † Dr. of medicine—Medical practitioner and Dental practitioner—often with the addition Professor or Teacher and Examiner; although they hardly have a conception of the different diseases of the Teeth or what remedies to have recourse to.

Many a one, through need, qualifies himself by reading practical and instructive guides or impelled by native integrity seeks good counsel from Dentists of known reputation, but the wished for information is either not imparted in the proper manner, or so miserably and unmethodically that they comparatively, become only competent through their own energy and industry.

The main point however, *the preservation of the Teeth* through solid and lasting fillings, one can only learn under a *capable instructor*, to be perfected by the opportunities of practice.

If I could show the letters I receive, which ever increase in number, as examples (and I am sure I must consider them as such,) it grieves me sorely that our schools for instruction fail in assisting such active assiduity. I have indeed on three occasions, when the parties had advanced

*** Dr. med—Praktischer Arzt. Praktischer Tahnarzt.**

**† In the German titles of Dr. they general precede the word Artz with praklisch—meaning practising his profession.**

to the second class in the gymnasium, accepted such as pupils; but when they had learned how to treat painful Teeth and then fill them with the different materials employed, to extirpate a nerve—to extract Teeth—to adjust Pivot Teeth—to take impressions and make Rubber work, etc., yet I have mentally felt that of *fundamental solidity* there could be no question, for Anatomy, Physiology, Analytic Enquiry and Microscopy could only be conversationally dwelt on and that without any preceding preparation.—It was imperfect, just piece work.

*Fundamental solidity* can only be acquired at an educational institution where the various branches can be inculcated *theoretically and practically*, by teachers who have devoted their time to the study of their particular departments.

*We have, it is true, in Germany dental teachers, but no proper Institutions where a dentist can be theoretically and practically educated.*

It is therefore for this reason that he who has the stimulus to accomplish something as a Dental practitioner, spares neither the time nor money necessary,—to receive in one of the eleven Dental Colleges of the United States, the educational culture he lacks. I know this, for I have frequently been applied to by such energetic young colleagues for letters of introduction.

Who will not find in these facts the surest evidence that our German Dental Schools cannot satiate that thirst for knowledge which the dental candidate expects? The majority even of these return after a half year's sojourn at an American Dental College * as *perfect dental operators*, for treating diseases of the teeth as well as filling them with gold.

(2) The *positive side* of how to bring up Dentists, I find much more difficult to treat, for even in America, the land of dental nurseries, the Reformers have not as yet discov-

* Notice that this verifies my statement in the commencement of this paper. W. R P.

ered the vital means of reaching their desire. Still the question should meet with an easier solution in Germany, as our excellent preparatory schools, viz. the Gymnasial, endows each with a well filled school bag that fits him for universal education, which is not the case in America, where many aspirants for medical as well as dental diplomas cannot even write correctly their own mother tongue.

Consequently, with us, Jewelers, Watch makers and such tradesmen should only be permitted to enter a dental educational institution when through attendance at a Gymnasium, (high school) they by the study of Latin could prove a scientifical ground work. To adhere to this in all cases might prove for one aspirant or another very inconvenient, but, a rule must exist and exceptions to every rule are admissable.

That the code of a *scientific ground work* must be adhered to is proven by the facts—that the best works written on general and special subjects of Dental science do not emanate from the Mechanical dentists, etc. but principally from qualified Dentists and then medical men and surgeons, with fundamental medical education.—It consequently follows that, a *foundational, scientifical, medical education is beyond every thing necessary, if we wish to be regarded not as superficial but as Dentists of sterling worth.*

But a farther important question is the following. Is one, who as a Dentist can write a very scientific production, also a practical Dentist? No. For he may not understand how to perform lasting operations through proper solid fillings.

He who wishes to gain celebrity as a Dental practitioner must add to his scientific knowledge, the manipulation and dexterous skill necessary to make faultless Gold fillings, otherwise he will ruin more teeth than he can conscientiously answer for, and does not earn the right to call himself a Dental practitioner—but to learn this; before all, comes the need of *establishing independent practical educational institutions for Dentists*;—where able technical instruction, necessary to the proper manipulation of difficult cavities, can be imparted. The large number of enquiries, that I receive, in which honorable practitioners admit their lack of skill and pray to be informed where and how the defect can be remedied, is to me the best proof that in none of our existing German schools, wherein lectures on "*all departments of Dentistry*" are held, do we possess the proper instructors for Operative work or filling Teeth. What avails the person who wishes to educate himself for practise, that he has so thoroughly studied the Anatomy, Physiology, Microscopy and Therapeutics of the Teeth, so as to teach and write books on these branches; when it becomes the question of preserving a painful, carious tooth, knows naught but how to apply a palliative remedy—order a leech—pressing a gutta-percha or amalgam filling, or extract that which treated by an able practitioner could be probably still retained for 10 to 20 years. Our *dental specialty* embraces such an extensive field that in establishing an Institution, six professors at the very least are absolutely necessary.

The writer then advances the branches necessary and what apertains to the department of each chair, which results in:

1 Anatomy and Physiology.
2 Chemistry and Metallurgy.
3 Dental Pathology and Therapeutics.
4 General and Oral Surgery.
5 Practical course of Operative Dentistry.
6 Mechanical Dentistry, in their varied relations to general practice, dwelling on the advantages of clinical demonstration—in fact the curriculum is similar to that of the U. S. Dental colleges—if any thing embracing a wider field. This is to be preceded by what we would term a high school education—would suffice for a commencement and be sure to bear good fruit; but he would not stop here. Later when the system would be firmly established, higher ground still should be taken and the candidate be required to be an M. D. before he became a D. D. S.—He then dives into ways and means, favoring connections with medical colleges, for the beginning. I have given the pith of his words, which express the feelings of the educated Dentist of Europe. Progress is up in arms and let us hope, that the strong hand of education may speedily stamp out the evil effects of charlatanism.—*Canada Jour. of Dental Science.*

